# DNA Methylation Alterations in Blood Cells of Toddlers with Down Syndrome

**DOI:** 10.3390/genes12081115

**Published:** 2021-07-23

**Authors:** Oxana Yu. Naumova, Rebecca Lipschutz, Sergey Yu. Rychkov, Olga V. Zhukova, Elena L. Grigorenko

**Affiliations:** 1 Vavilov Institute of General Genetics RAS, 119991 Moscow, Russia; sergey.rychkov@gmail.com (S.Y.R.); zhukova@vigg.ru (O.V.Z.); 2 Department of Psychology, University of Houston, Houston, TX 77204, USA; Rebecca.Lipschutz@times.uh.edu; 3 Department of Psychology, Saint-Petersburg State University, 199034 Saint Petersburg, Russia; 4 Department of Molecular and Human Genetics, Baylor College of Medicine, Houston, TX 77030, USA

**Keywords:** Down syndrome, infants and toddlers, trisomy 21, DNA methylation, Illumina 450K Human Methylation Array

## Abstract

Recent research has provided evidence on genome-wide alterations in DNA methylation patterns due to trisomy 21, which have been detected in various tissues of individuals with Down syndrome (DS) across different developmental stages. Here, we report new data on the systematic genome-wide DNA methylation perturbations in blood cells of individuals with DS from a previously understudied age group—young children. We show that the study findings are highly consistent with those from the prior literature. In addition, utilizing relevant published data from two other developmental stages, neonatal and adult, we track a quasi-longitudinal trend in the DS-associated DNA methylation patterns as a systematic epigenomic destabilization with age.

## 1. Introduction

Down syndrome (DS) is one of the most common chromosome abnormalities. According to the World Health Organization, the incidence of DS is between one and 1000 live births in the world population. The syndrome is caused by the presence of additional genetic material of chromosome 21 and is associated with physical growth delays, intellectual disability, and other developmental and physical impairments and comorbidities. More than 80 clinical DS phenotypes have been defined that may not be explained merely by the triplication of genes located on chromosome 21. Moreover, the expression of these genes may remain unaltered in DS, despite the expected effect of “gene dosage” [1]. To date, most genomic studies tend to associate this diversity of DS clinical phenotypes with a destabilization of the entire genome due to additive influences of the trisomy on related genes within a network or pathway that result in alterations in gene expression and the mechanisms of its control, including DNA methylation.

A growing body of research on DNA methylation in DS consistently reports trans epigenetic effects, where the presence of an additional chromosome 21 affects methylation on other chromosomes. Chromosome 21 contains epigenetic modifier genes, such as DNA methyltransferase *DNMT3L*, involved in the de novo methylation process whose extra-activity due to a “dosage effect” may contribute to genome-wide epigenetic dysregulation [2,3]. In addition, an overdosage of transcription factors located on chromosome 21, such as *RUNX1* binding to the core element of many enhancers and promoters, may disturb the genome-wide pattern of the chromatin structure and DNA methylation [3,4]. Accordingly, significant genome-wide perturbations in DNA methylation in DS, in comparison to matched controls, have been revealed in various cells and tissues, such as placenta tissue [5], blood cells [6,7,8,9], buccal epithelial cells [10], and neural tissue [4,11,12,13]. Genome-wide DNA methylation alterations have been detected in individuals with DS at different developmental stages throughout the lifespan—specifically, at the prenatal [11,14] and early postnatal stages [7,8,9] and in adulthood [6,10,12]. A recent meta-analysis provided evidence that a number of loci and genes might be consistently implicated in epigenetic mechanisms of DS across tissues and developmental stages [3]. At least 25 such pan-tissue genes predominantly hypermethylated in DS have been identified [3] that are involved in a broad spectrum of biological processes and, above all, in transcription regulation, signal transduction, and neurodevelopment. Consequently, the current literature reporting on systematic genome-wide epigenetic perturbations in DS has shown that DNA methylation alterations may be implicated in multiple developmental impairments and disease phenotypes in DS, such as defects in immune system development and hematopoiesis [8], altered neurodevelopment and brain function [12], and cognitive impairments [10], among others.

In this study, filling a gap in the data on epigenetic perturbations in individuals with DS of different age groups, we report on the genome-wide DNA methylation alterations in blood cells of children with DS at a critical stage of development—in infancy and toddlerhood, from 0.5 to 4.5 years of age. We discuss these study results in the context of the extant literature on DS-related epigenetic alterations, emphasizing the high level of consistency and reproducibility of the findings. In addition, by engaging relevant empirical data for newborns [8] and adults [6] with DS, we track a quasi-longitudinal trend in the age dynamics of DS-associated DNA methylation alterations in blood cells.

## 2. Materials and Methods

### 2.1. Participants

The study participants were 34 children between the ages of 0.5–4.5 years: 17 with Down syndrome (DS) and 17 typically developing (TD) children. The participants were ethnically homogeneous and of Eastern Slavic origin. The comparison groups, DS and TD, were matched in terms of gender ratio and children’s age. Approximately 40% in each group were females; participant age was not significantly different between the groups (Welch’s unequal variances *t*-test *p*-value = 0.91): DS Mean age = 33.88 ± 16.22 mos., six girls, and TD Mean age = 33.35 ± 11.28 mos., seven girls. The individual data on the children’s demographics are shown in Supplementary Table S1. Additionally, given that the children from both the DS and TD groups were recruited from state-run orphanages in St. Petersburg, Russia, they all shared the same living environment and received the same care. For the children with DS, the syndrome and the type of chromosomal abnormality were confirmed by cytogenetic analysis. All children with DS were characterized by trisomy 21 and had a karyotype 47,XX,+21 or 47,XY,+21.

### 2.2. DNA Methylation Profiling and Data Processing

Genomic DNA was isolated from peripheral blood using the FlexiGene^®^ DNA Kit (Qiagen, Hilden, Germany), per the manufacturer’s instructions. Approximately 600 ng of DNA was used for bisulfite conversion using the Zymo Research EZ DNA Methylation Kit (Zymo Research, Irvine, CA, USA). After bisulfite treatment, 160 ng of DNA was applied to the Illumina Infinium HumanMethylation450 array (HME450), as per the manufacturer’s protocols (Illumina, San Diego, CA, USA). The microarray contains genome-wide probes for 485,577 methylation sites, or CpG sites, at a single-nucleotide resolution. The Illumina iScan system was used to scan the 450 HME microarrays.

For the microarray data processing, the analysis pipeline, provided by the Minfi R package, was used [15]. The raw microarray data were preprocessed/normalized using a stratified quantile normalization procedure implemented by the Minfi preprocessQuantile function. The microarray data were subjected to quality control (QC). First, for the sample-specific QC, the minfiQC function was applied. All samples met the main QC criteria: the log-median intensity of the methylated and unmethylated signals was >11 [15]. Second, a total of 3,038 probes with missing values or a detected *p*-value greater than 0.05 in more than 10% of the samples were removed. The probes were filtered; the probes located on sex chromosomes and the probes having a polymorphic variant at the target CpG (with a minor allele frequency, MAF > 0.05) were removed. The remaining 461,258 CpGs were involved in further analysis. The relative DNA methylation measurements (beta-values) were corrected for the individuals’ blood cell-type composition to eliminate the effects of individual cellular heterogeneity and potential between-group differences in the cell-type distributions on the results of the differential methylation analysis. For that, an estimated cell-type composition was obtained from the methylation dataset using a prediction algorithm utilizing a reference dataset of six different types of white blood cells [16] implemented in the FlowSorted.Blood.450k package [17].

### 2.3. Differential Methylation Analysis

CpG sites with DNA methylation levels significantly different between the DS and TD groups were identified using moderated empirical Bayes *t*-tests with Benjamini-Hochberg corrections, implemented in the Limma R package [18]. Significant sites (at *p*_adj_ < 0.05) were filtered, and the CpGs that had an intergroup difference in the mean beta-value of at least 1.2-fold change [19] were defined as differentially methylated positions (DMPs). The bump-hunting algorithm [20] implemented in the Minfi package was applied to discover clusters of CpGs located within the same genomic element with unidirected significant (at a *p*_adj_ < 0.05) differences in the mean methylation levels between the comparison groups (DS vs. TD). These CpG clusters were defined as differentially methylated regions (DMRs).

Genomic annotation of the differentially methylated genes (DMGs) was performed using the HME450 microarray manifest and the UCSC databases [21]. For the analysis of the DMG set enrichment in the Gene Ontology (GO) [22,23] terms and the Human Phenotype Ontology (HPO) [24] terms, the ShinyGO tool [25] was used. False discovery rate-adjusted *p*-values were obtained whenever appropriate to control for multiple testing.

### 2.4. Analysis of Differential Methylation across Age Groups

To examine the potential longitudinal dynamics of DS-associated alterations in DNA methylation throughout the lifespan, we performed a quasi-longitudinal analysis comparing the results of three relevant cross-sectional studies (Table 1). In addition to the current study results, we included data from a recently published case–control study in newborns with DS [8] and data from a family-based study [6] comparing DS probands with non-DS siblings in an older cohort of DS individuals aged 12–43 years (mean age of 26 years), who were defined here as adults. All three studies relied on similar techniques for DNA methylation profiling; they used a version of the Illumina Infinium methylation microarray. However, besides the differences in the participants’ ages, these studies engaged cohorts of diverse ancestry, utilized different study designs, and used various approaches and analytical pipelines to detect DS-associated differentially methylated patterns.

## 3. Results

### 3.1. Blood Cell-Type Count in DS vs. TD Toddler Groups

Hematological research has reported defects in the immune system in DS related to both abnormalities in blood cell morphology and function and the altered prevalence of different blood cells’ subpopulations [28,29,30]. Considering that DNA methylation is tissue- and cell-specific, the differences in the cell-type proportions could bias the results of the differential methylation analysis when comparing DNA methylation in whole blood from children with and without DS. Since blood cell-type counts were not available for the studied cohort, we used a well-established algorithm to recover the blood cell-type compositions from DNA methylation data [31]. The results of the between-group comparisons of the cell-type compositions are summarized in Table 2; for the individual data, see Supplementary Table S1. A significant difference was obtained in the distribution of NK cells in the DS compared to TD group (t = 2.406, df = 31.78, *p* = 0.022). Additionally, despite a lack of statistical significance, we observed a decrease in the number of CD4+ lymphocytes and an increase in CD8+ cells in DS, leading to the CD4+/CD8+ ratio of 0.87, which is lower than the normal ratio range of 1–4. These findings are consistent with previously reported alterations in the blood cell compositions related to DS, such as a decrease in B lymphocytes and CD4+ T lymphocytes and an increase in NK cells [32,33,34].

### 3.2. Differentially Methylated Positions (DMPs) in DS vs. TD Toddler Groups

**Figure 1:**
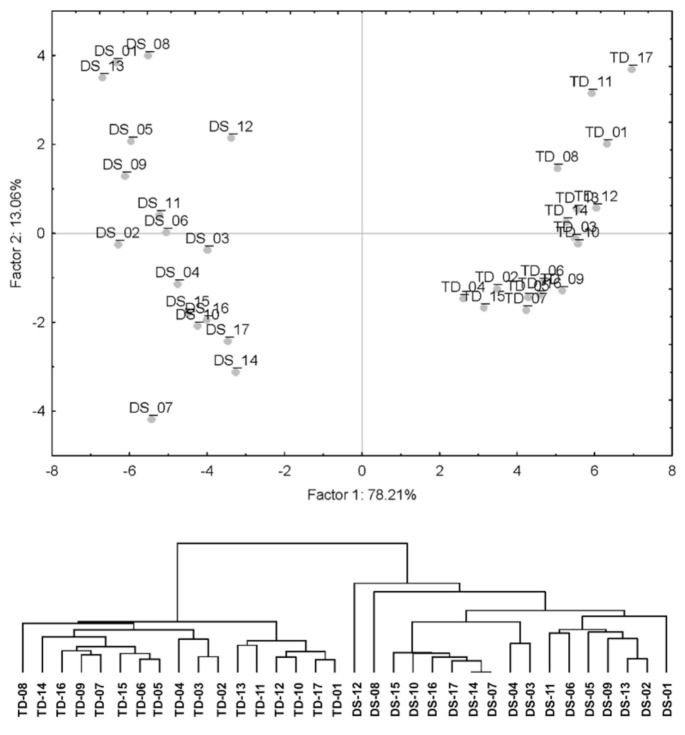
Clustering analysis of children with and without DS based on the methylation levels of 4806 DMPs: PCA plot based on the correlation matrix (**top**), and a hierarchical tree constructed using the Manhattan distances and the Ward clustering algorithm (**bottom**). The plots show that the toddlers with and without DS are grouped into separate remote clusters based on the DMP methylation profiles.

Differential methylation analysis revealed 4806 CpGs with a significant (at a *p*_adj_ < 0.05) difference in the average methylation level (beta-value), with an at least 1.2-fold change between children with and without DS. The distribution of the beta-values of these differentially methylated positions (DMPs) across the individuals and comparison groups is represented in Supplementary Table S2. Most of the DNA methylation differences in DS, compared to TD, were hypermethylation events: of 4806 DMPs, 3921 (or 82%) were hypermethylated, and 885 CpGs (or 18%) were hypomethylated in DS. The results of the principal components analysis (PCA) and hierarchical clustering analysis showed that the methylation profiles of the detected DMPs have enough power to reliably discriminate the comparison groups of children into distinct clusters: DS and TD (Figure 1).

**Figure 2:**
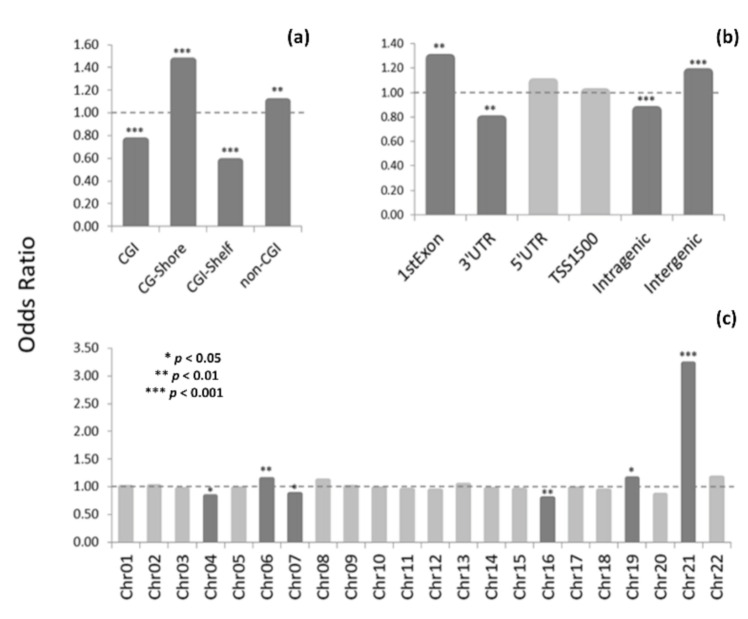
Plots depicting a comparison of the genomic distributions of DMPs—in the context of their relation to CGIs (**a**), location within a gene region (**b**), and chromosomal localization (**c**)—with the corresponding genomic distributions of the HME450 probes using the odds ratio (OR). The OR represents a difference between the observed frequency of a genomic region in the DMP set and the expected frequency of the region estimated based on the HME450 probe content. The OR significance levels estimated based on Fisher’s exact ratio test are marked by asterisks.

**Figure 3:**
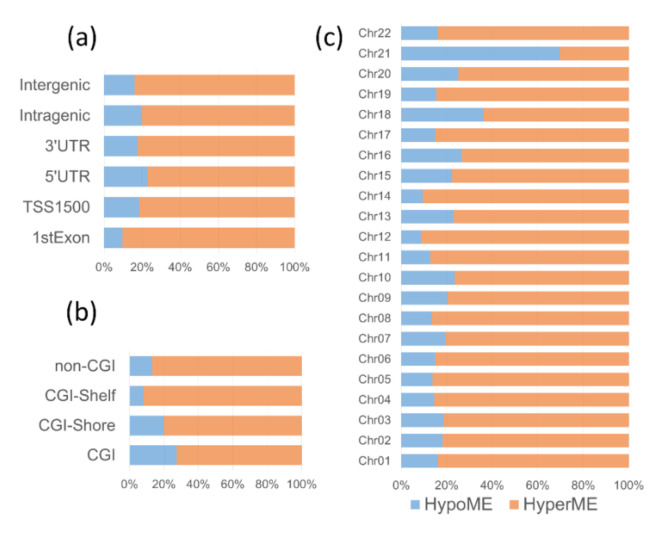
The percentage of the hypermethylated (HyperME) and hypomethylated (HypoME) DS-associated DMPs by gene regions (**a**), CpG islands, CGIs (**b**), and autosomes (**c**).

Comparing the DMP distributions in the genomic contexts (Table S3) to those of the probes contained in the HME450 array showed a nonrandom localization of DS-associated DNA methylation alterations (Figure 2). Concerning CpG-islands (CGI), there was significant enrichment in the DMPs located in non-CGI regions and CGI-flanking regions (CGI shores), whereas DMPs in CGIs and CGI shelves were underrepresented (Figure 2a). In regard to gene regions, the DMPs were significantly overrepresented in the first exons and intergenic regions and underrepresented in the 3′ untranslated regions (3′UTR) and intragenic regions (Figure 2b). The enrichment in the DMPs located in certain genomic regions may indicate both a systemic characteristic of the DS-related changes in methylation and a particular “sensitivity” of these genomic regions to the DS-associated methylation changes. Concerning the chromosomal distribution, DMPs were observed across all autosomes (Figure 2c), with a remarkable overrepresentation of the DMPs located on chromosome 21 (OR = 3.3462; 95% CI = 2.8274–3.9604; *p* < 0.0001). Notably, hypermethylation events in DS were consistently predominated across all genomic regions (Figure 3a,b) and across all autosomes except chromosome 21 (Figure 3c).

**Figure 4:**
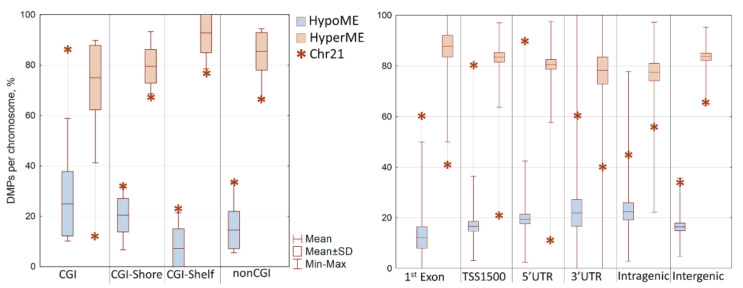
Plots depict the percentage of hypermethylated and hypomethylated DMPs of chromosome 21 relative to the corresponding characteristics of other autosomes; the percentages across different regions—CGIs (**left**) and genic regions (**right**)—are shown. The values for chromosome 21 are marked by asterisks. Boxplots show the statistics for the distribution of the percentage of hypermethylated and hypomethylated DMPs—the mean value, maximum, and minimum—for all autosomes except chromosome 21.

The DMPs on chromosome 21 showed an opposite methylation profile to the other autosomes; most of the DMPs on chromosome 21 were hypomethylated—69.6% compared to 18.3% ± 6.4% of hypomethylated DMPs on the other autosomes (Figure 3c). Considered separately, the percent distributions of hypermethylated and hypomethylated DMPs by genomic regions and chromosomes also indicate a distinctive methylation profile of chromosome 21, compared to the other autosomes (Figure 4). On chromosome 21, the hypomethylated CpGs in DS are remarkably predominant within CGIs (69.4% hypomethylated DMPs vs. 24.4% ± 6.9% on other autosomes), especially those related to gene promoters: 5′UTRs (88.6% vs. 19.5% ± 8.6%) and regions upstream of the transcriptional start sites (TSS1500; 80.0% vs. 16.5% ± 8.7%).

### 3.3. Differentially Methylated Genes (DMGs) in DS vs. TD Toddler Groups

**Figure 5:**
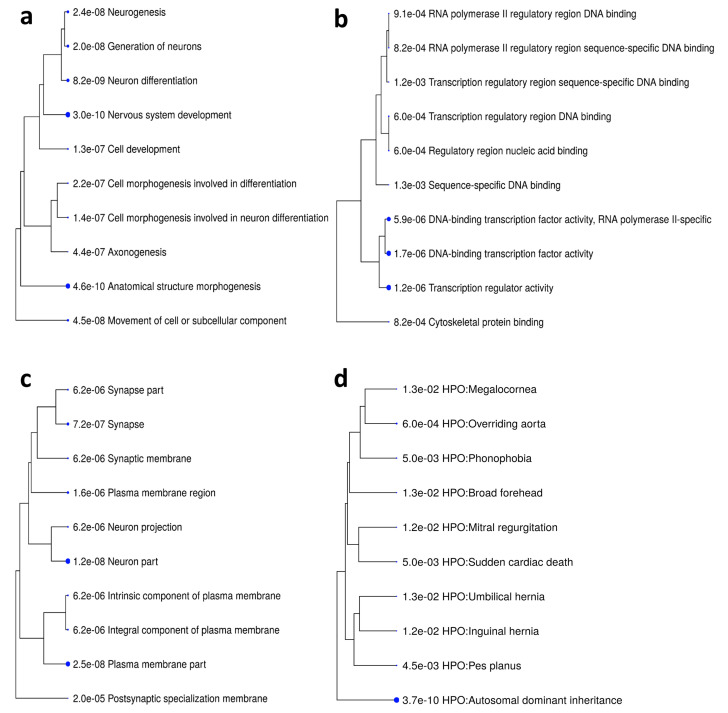
Hierarchical clustering trees summarizing the results of the Gene Ontology—GO:Biological Process (**a**), GO:Molecular Function (**b**), and GO:Cellular Component (**c**)—and Human Phenotype Ontology (**d**) overrepresentation tests performed for the set of genes differentially methylated (DMGs) in DS (Table S4). The trees were constructed using ShinyGO tools [25]. The top 10 GO terms overrepresented in the DMGs set ranged by the enrichment *p*-value are shown. Functional categories are clustered based on the number of shared genes; bigger dots indicate more significant *p*-values.

The genomic annotation of DMPs revealed that 1,998 of 4,806 differentially methylated CpGs might be related to a gene promoter; namely, they are located in a gene first exon, 5′UTR, and/or in the 200–1500-bp region upstream of the TSS (Transcription Start Site). Cumulatively, 1238 such genes with DS-specific methylation signatures in their promoters were identified and defined as differentially methylated genes (DMGs); the list of DMGs is provided in Supplementary Table S4. To analyze the biological processes and pathways that these DMGs control and the clinical manifestations they are known to be involved in, we performed several tests of gene set enrichment of the particular GO and HPO terms. The results of these tests are presented in Supplementary Table S5; the top 10 terms overrepresented in the DMGs set are shown in Figure 5.

As shown in Figure 5, the GOs most significantly overrepresented among the DMGs are those involved in the transcription regulation (Figure 5b) related to developmental processes, particularly, the central nervous system development and function (Figure 5a,c). Concerning the potential association with particular phenotypic features, the DMGs have been linked to a broad spectrum of diseases and phenotypic abnormalities (most of which are assigned to a group of autosomal-dominant disorders) that have a high prevalence in DS. Thus, the HPOs listed in Figure 5d include phenotypic characteristics often associated with disorders and conditions in DS, including megalocornea, which is often associated with ocular disorders in DS [35], pediatric hernias [36], cardiovascular system conditions [37], and others.

### 3.4. Differentially Methylated Regions (DMRs) in DS vs. TD Toddler Groups

**Figure 6:**
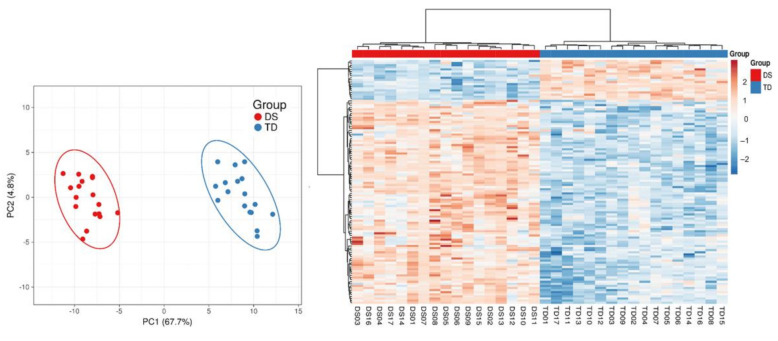
PC plot and heatmap depicting the clustering of toddlers with and without DS (the heatmap’s columns) based on the methylation levels of 115 DMRs (the heatmap’s rows).

An analysis of the differentially methylated CpG clusters using the bump-hunting algorithm, following the adjustment for the variation in cell-type proportions, revealed 115 differentially methylated regions (DMR) in toddlers with DS (Table S6). As shown in Figure 6, DMR-based clustering reliably separated DS from TD toddlers. Remarkably, consistent with the DMP methylation pattern, most regions (96 of 115 DMRs or 83.5%) were hypermethylated in DS (see the heatmap in Figure 6). The genomic annotation indicated that the 115 DMRs overlapped 111 unique genes across all the autosomes, and 57 DMRs were located within a region related to a gene promoter—the 5′UTR or TSS1500 region (Table S6).

The top 19 genes that demonstrated the most profound DS-associated methylation differences in the promoter region (a mean beta-difference or delta-beta value over 0.15) are shown in Table 3. Most of these genes are known to be involved in the manifestation of various clinical phenotypes [38], such as metabolic diseases (*CPT1B* and *BLVRA*); hemorrhagic diseases (*RUNX1* and *GP6*); and neuronal disorders (*KCNAB3*, *FAM179B*, *CCDC60*, *GRM6*, and *PRDM8*), among others (Table 3). In the context of primary gene functions, among those 19, several genes and related functional categories should be highlighted: genes involved in catabolic processes (*DAPL1*, *BLVRA*, *DPEP1*, and *CPT1B*); genes controlling cell motility and migration (*DPEP1*, *GP6*, and neuronal navigator *NAV1*); and transcription factors involved in a broad spectrum of developmental processes—in particular, in anatomical and cellular development (*PRDM8*, *FLI1*, and *RUNX1*), and hematopoiesis (*FLI1* and *RUNX1*). It is important to note, in recent epigenome-wide association studies in blood cells of newborns with trisomy 21, the last two genes, *FLI1* and *RUNX1*, have shown the most significant DS-associated differential methylation [8,9].

### 3.5. DS-Specific DNA Methylation Pattern in Blood Cells throughout the Lifespan

To explore the consistency of our findings against previously published evidence on DS-related epigenetic alterations, and to examine the potential longitudinal dynamics of such alterations throughout the lifespan, we performed a quasi-longitudinal analysis. In addition to our results on differential methylation in the blood of toddlers with DS, we included relevant data from newborns [8] and adults [6] with DS; for detailed characteristics of these datasets, see the Section 2, Table 1. A summary of the results of the differential methylation analysis for the three studies is shown in Table 4. Despite the differences in the analysis pipelines, study designs, and the genetic backgrounds of the participants across the three datasets, we found a high level of consistency between the findings and considerable overlap in the DS-associated methylation patterns detected in the three association studies.

**Figure 7:**
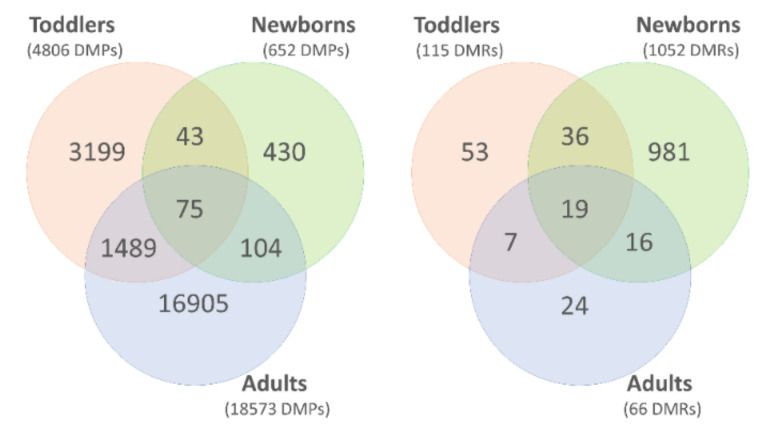
The distribution and overlapping of differentially methylated positions (DMPs; **left**) and differentially methylated regions (DMRs; **right**) identified in three epigenome-wide association studies in the individuals with Down syndrome of different ages: newborns [8], toddlers (current study), and adults [6].

**Figure 8:**
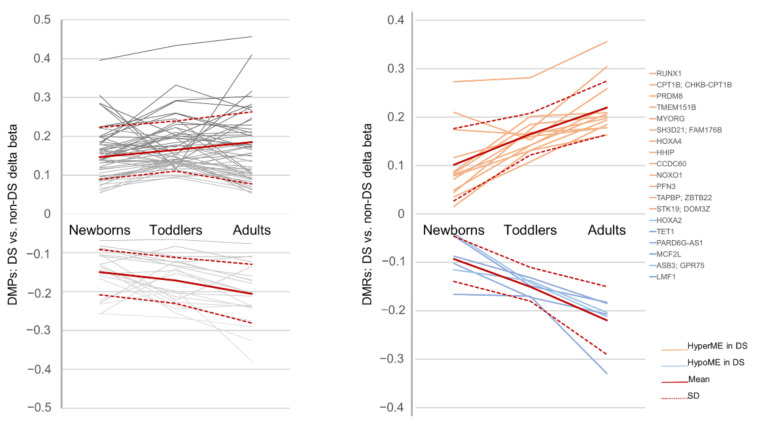
Plots depict DS-associated methylation signals—75 DMPs (**left**) and 19 DMRs (**right**)—consistently detected in the blood cells of individuals with DS across three age groups. The *Y*-axis shows the mean delta-beta of DS vs. non-DS individuals; the positive and negative values of the mean delta-beta correspond to the hypermethylation and hypomethylation events in DS, respectively. The delta-beta average by age group marked by the red line indicates the main trend of longitudinal change in DS-associated differential methylation.

At the single methylation site level, we identified over 1.5 K CpG sites showing significant differential methylation in at least two age groups of individuals with DS, compared to controls, and 75 DMPs were found in all three age groups (Figure 7). The list of the 75 overlapping DMPs is provided in Supplementary Table S7. They all showed identical differential methylation patterns across the age groups—either hypomethylation or hypermethylation in DS—and most of them (54 of 75 DMPs or 72%) were hypermethylated (Table S7 and Figure 8). At the level of the CpG clusters, 131 DS-associated DMRs were detected in at least two comparative studies (Table S8), and 19 of them were identified in all three age groups (Figure 7). Similar to the previous observation, the differential methylation patterns of the 19 overlapping DS-associated regions were identical across the three age groups, and most of the DMRs (13 out of 19 DMRs or 68%) were hypermethylated in DS (Table 4 and Figure 8). Genomic annotations of the 19 DMRs showed that a number of them are related to the promoter regions of critical developmental genes: a morphogen *HHIP* and the regulators of the transcription factors *HOXA2*, *HOXA4*, *TET1*, *PRDM8*, *ZBTB22*, and the *RUNX1* mentioned above, which shows the most profound DS-associated hypermethylation (Table 5 and Figure 8).

**Figure 9:**
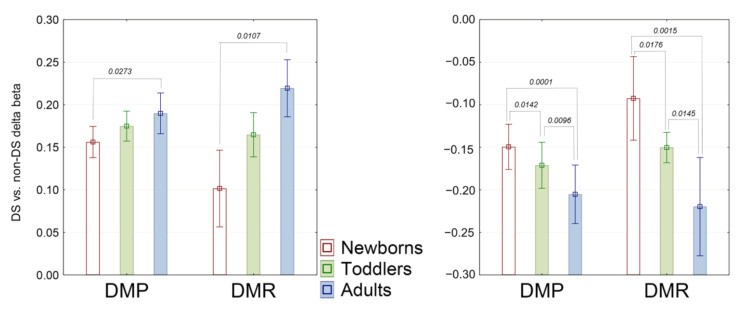
A comparison of the means of the delta-beta values of the DS-associated DNA methylation signals—differentially methylated positions (DMP) and regions (DMR)—across the three age groups. Whiskers depict the confidence interval for each mean. The left and right panels represent the data for the hypermethylated and hypomethylated DMPs and DMRs, respectively. Only statistically significant *p*-values (*p* < 0.05) for the paired *t*-tests are shown.

To explore the potential dynamics in DS-associated methylation patterns throughout the lifespan, we tracked the differential methylation levels of both CpG sites and genomic regions across three developmental stages: neonatal, early childhood, and adulthood. The plots in Figure 8 show the dynamic profiles of the methylation differences as the mean delta-beta values between individuals with DS and relevant controls for the 75 DMPs and 19 DMRs, along with a generalized profile derived as the delta-beta averaged by age group. Despite relatively small sample sizes, a comparison of the delta-beta means across the three age groups, based on t-tests, revealed statistically significant differences for the hypomethylated DMPs and DMRs in all pairwise comparisons and significant differences between newborns and adults for the hypermethylated DS-associated signatures (Figure 9). As can be seen from Figure 8, regardless of the marker system describing the methylation patterns, the main trajectory of the longitudinal change in the DS-associated methylation alterations is an increase in both hyper- and hypomethylation with the ages of individuals with DS.

## 4. Discussion

To compare the DNA methylation profiles in the blood cells of young children with Down syndrome to those of typically developing peers, we examined the DS-associated differential methylation patterns considered at the level of single CpG sites (differentially methylated positions, DMPs) and their clusters (differentially methylated genomic regions, DMRs). Summarizing the main finding of this analysis, we should point out a few observations. First, we observed significant genome-wide alterations in DNA methylation: over 4000 DMPs and 115 DMRs were identified across all autosomes in the blood cells of toddlers with DS. This finding is consistent with prior empirical literature, which has provided evidence of genome-wide perturbations in the DNA methylation of different cells and tissues of individuals with DS, such as blood cells [6,7,8,9], buccal epithelial cells [10], and neuronal tissue [11,12,13].

Second, most DS-associated DNA methylation differences found in children with DS were hypermethylation events: 82% of the DMPs and 83.5% of the DMRs were hypermethylated. Notably, there was some inconsistency in the findings on global methylation effects due to trisomy 21. Data on the prevailing global methylation pattern are different across studies using diverse tissue types, engaging individuals with DS at different developmental stages and utilizing various methylation profiling techniques and analytical methods; yet, most commonly, hypermethylation effects have been reported. The mechanistic hypotheses regarding trans-hypermethylation in DS due to trisomy 21 are well-articulated [3]; they suggest that the hypermethylation may be due to an increased dosage of chromosome 21 genes involved in methylation pathways per se (e.g., *SLC19A1*, *FTCD*, *CBS*, *PRMT2*, and *DNMT3L*) and/or due to abnormal patterns of particular transcription factors binding site (e.g., sites for *CTCF* and *RUNX1*) occupancies. Importantly, a trend for global hypermethylation has been consistently observed in DS fetal and adult brain tissue [4,11,13,39], as well as in placenta tissue [5,14]. Thus, almost equal proportions of hyper- and hypomethylated events have been found in the blood of newborns with DS [7,8] and in the peripheral T lymphocytes of adults with DS [4]. Moreover, practically all cross-tissue signatures in DS-associated methylation patterns have been related to hypermethylation events. Recent studies have reported on 25 pan-tissue genes with an altered methylation pattern in DS [3]; 24 of these genes were hypermethylated in DS, whereas hypomethylation in DS tends to be related to tissue-specific methylation patterns [3,9]. The comparison of differential methylation in the blood cells of individuals with DS of three age groups allowed us to speculate that such a predomination of hypomethylated signatures in DS may constantly increase with age. As we observed, the percentage of hypermethylated CpG sites and genomic regions remarkably increases from 48% in newborns to 60–80% in children and adults with DS (see Table 4). Given some limitations of our cross-age comparison due to the different analytical pipelines used for the detection of DS-associated methylation in each age group, this assumption requires further confirmation from relevant longitudinal research or meta-analyses.

Third, we noticed that chromosome 21 exhibits a differential methylation pattern distinct from those of other autosomes. Specifically, chromosome 21 was significantly enriched in CpG sites differentially methylated in toddlers with DS. Additionally, in contrast to other autosomes whose DS-specific methylation patterns include the predominant hypermethylation of single CpGs located within first exons and intergenic regions, chromosome 21 presents significant hypomethylation, especially of the sites located within CpG islands related to promoter regions. These findings are consistent with the results reported in several empirical studies, which also found a distinct “hypomethylated” pattern of chromosome 21 in DS [8,11,12]. Remarkably, it has been reported in studies utilizing various tissues, but they all focused on early developmental stages, such as those in the developing cortical tissue [11,12] and in the blood cells of newborns [8]. This observation allows us to speculate that chromosome 21 may undergo more significant hypermethylation changes over the lifespan in individuals with DS as a compensatory response to attenuate the overexpression of chromosome 21 genes. In this case, the originally “hypomethylated” profile may be substantially diminished in adult individuals with DS. Further research, longitudinal or quasi-longitudinal, focused on the lifespan dynamic changes in DS-associated methylation signatures could help verify this observation. Consistent with the relevant published research focused on DS-associated methylation alterations [6,8,10,12,40,41], we provided further evidence that a significant perturbation in the methylation of a number of genes may be involved in the manifestation of multiple developmental impairments, including intellectual disability, and other disease phenotypes in DS. Thus, we showed that a substantial number of genes involved in transcription regulation and genes controlling a wide spectrum of developmental processes undergo significant DS-associated methylation alterations. In the context of potential impact on phenotypic features, we found that such methylation disturbances occur in the genes known to be associated with metabolic diseases, hematopoietic disorders and myeloid leukemias, cardiovascular system conditions, and neuronal disorders, all of which have a high prevalence in individuals with DS.

Comparing our findings with the relevant published data on differential methylation in the blood cells of newborns [8] and adults [6] with DS, we identified 75 CpG sites and 19 CpG clusters (or genomic regions) that consistently showed DS-associated methylation signals in all three age groups. Most of them—72% of the methylation sites and 68% of the regions—were hypermethylated in DS, compared to the age-matched controls. Based on the differentially methylated regions overlapping across the age groups, we highlighted a number of critical developmental genes consistently demonstrating DS-associated changes in the methylation of promoter regions: a morphogen *HHIP* and regulators of the transcription factors activity: *HOXA2*, *HOXA4*, *TET1*, *PRDM8*, *ZBTB22*, and *RUNX1*.

One of these genes, *RUNX1*, located on chromosome 21, showed the most profound hypermethylation in DS across all age groups. Prior literature assigned *RUNX1* to a group of genes that exhibit the pan-tissue hypermethylation in DS [3,12]. In addition, the primary role of *RUNX1* hypermethylation, following its transcriptional excess due to trisomy 21, has been suggested to be a driver of DS-related epigenome-wide dysregulation [9]. Tracking *RUNX1* differential methylation across three ages as a difference between the individuals with and without DS, we observed a remarkable increase in *RUNX1* hypermethylation with age; in terms of the delta-beta values, it increased from 0.27 to 0.28 in newborns and toddlers to 0.36 in adults. Similar to the distinct increase in the *RUNX1* hypermethylation with age in DS, we found that the entire DS-associated methylation pattern shows a tendency to increase differential methylation between individuals with and without DS with aging. Specifically, we observed a consistent increase in both types of DS-associated signals—hypermethylation and hypomethylation—from the neonate to the adult stage. Such exacerbating DS-associated methylome differences between individuals with and without DS with age may indicate an aggravation of trisomy-related destabilization of the epigenome during the lifespan, which, in turn, may be a part of the story described as accelerated epigenetic aging in Down syndrome [42].

In conclusion, here, we provide new data on genome-wide DNA methylation perturbations in blood cells of individuals with trisomy 21 from a previously understudied age group—infants and toddlers aged 0.5–4.5 years. Despite the main limitation of this study, a small sample size, we report findings that are highly consistent with and partially replicate the published evidence on systematic alterations in genome-wide DNA methylation in Down syndrome. Combining the data from this study with the published findings on differential methylation in blood cells in individuals with DS at two other developmental stages—neonatal and adult—allowed us to track a quasi-longitudinal trend in DS-associated DNA methylation patterns as a systematic aggravation of methylome destabilization with aging. To our knowledge, our study is one of the first attempts to trace an age-related trend in DS-associated DNA methylation patterns. We believe that further research utilizing a conventional longitudinal approach and/or involving larger cohorts of participants could be fruitful for understanding the age dynamics of trisomy-related epigenome perturbations and their potential role in developmental impairments and health problems in Down syndrome.

## Figures and Tables

**Table 1:** Three datasets involved in a quasi-longitudinal analysis of DS-associated DNA methylation alterations in blood cells.

	Muskens et al. 2021 [8]	Current Study	Bacalini et al. 2015 [6]
Age Group	Newborns	Toddlers	Adults
Age, y (range)	0	2.8 ± 1.4 (0.5–4.5)	26.3 ± 9.5 (12–43)
Ethnicity	Mixed: Whites, Blacks, Asians	Whites; East Slavs	Whites; Italians
DS Sample Size, *n*	198	17	29
Study Design	Case-Control	Case-Control	Family-based Case-Control
Methylation profiling	EPIC microarray	HME450 microarray	HME450 microarray
Differential methylation analysis	DMRcate [26] and comb-p [27]; EWAS correction for cell counts, sex, and ancestry	Minfi [15] and bump-hunting [20]; EWAS correction for cell counts and batch	MANOVA and ANOVA of the pre-clustered blocks of probes; EWAS correction for cell counts, sex, and batch

**Table 2:** The distribution of blood cell types estimated based on DNA methylation data in toddlers with Down syndrome (DS) and typically developing peers (TD) and statistics on the intergroup comparisons of the cell-type compositions; *p*-values < 0.05 marked by asterisks.

Cell Type	Group	Mean	SD	Welch’s *t*-Test			Mann–Whitney *U* Test		
				*t*-Value	df	*p*-Value	*U*-Value	Z-Score	*p*-Value
T cells CD8+	DS	0.1903	0.0287	0.234	30.64	0.817	128.5	0.534	0.596
	TD	0.1877	0.0356						
T cells CD4+	DS	0.1673	0.0448	−1.305	29.02	0.202	112.5	−1.09	0.281
	TD	0.1917	0.0625						
NK cells	DS	0.0919	0.0361	2.406	31.78	0.022 *	85.0	2.03	0.042 *
	TD	0.0608	0.0392						
B cells	DS	0.1371	0.0227	−1.970	25.72	0.059	84.0	−2.07	0.039 *
	TD	0.1587	0.0391						
Monocytes	DS	0.0610	0.0192	−0.918	29.08	0.366	113.0	−1.07	0.255
	TD	0.0683	0.0265						
Granulocytes	DS	0.3665	0.0490	0.983	25.85	0.335	113.5	1.05	0.294
	TD	0.3433	0.0836						

**Table 3:** Top differentially methylated regions (DMRs) in the blood cells of toddlers with Down syndrome compared to typically developing peers; the DMRs are ordered by the mean methylation level difference (mean delta-beta value) in DS.

DMR Position (GRCh37/hg19)	CpGs, *n* (Cluster, *n*)	Mean Delta-Beta	*p*_adj_	Gene Symbol	Gene Name	Gene Function and Associated Phenotype
chr21:36258423-36259797	7 (7)	0.2812	1.03 × 10^−4^	*RUNX1*	Runt-related transcription factor 1	Transcription factor; Hematopoiesis; Hemorrhagic diseases; Blood platelet diseases
chr14:45431685-45432516	6 (16)	0.2053	8.57 × 10^−3^	*FAM179B*	TOG array regulator of axonemal microtubules protein 1	Primary cilia organization; Joubert syndrome, Spinocerebellar ataxia
chr16:89690088-89690262	2 (9)	0.1897	2.40 × 10^−3^	*DPEP1*	Dipeptidase 1	Kidney membrane enzyme; Glutathione metabolism; Blau syndrome, Glutamate-cysteine ligase deficiency
chr1:201618030-201619787	8 (16)	0.1827	1.71 × 10^−3^	*NAV1*	Neuron navigator 1	Neuronal migration and axon guidance; Episodic pain syndrome, Long qt syndrome
chr4:186732837-186733060	7 (9)	−0.1810	4.57 × 10^−3^	*SORBS2*	Sorbin and SH3 domain-containing protein 2	Adapter protein; Signaling complexes assembling; Hypotrichosis-13, Spheroid body myopathy
chr7:43803803-43804002	2 (2)	0.1794	2.86 × 10^−3^	*BLVRA*	Biliverdin reductase A	Catalyze; Biliverdin to bilirubin conversion; Hyperbiliverdinemia, Cholestasis
chr19:55549590-55549746	3 (10)	−0.1767	1.37 × 10^−2^	*GP6*	Platelet glycoprotein VI	Collagen-induced platelet adhesion and activation; Bleeding disorder platelet-1
chr5:176827082-176827697	5 (7)	0.1747	1.37 × 10^−2^	*PFN3*	Profilin-3	Regulation of actin cytoskeleton, Ras signaling pathway
chr21:44898090-44898206	3 (7)	−0.1699	1.49 × 10^−2^	*C21orf84*	Long Intergenic Non-Protein Coding RNA 313	Long noncoding RNA; Lung cancer, Brain glioma
chr1:170115042-170115351	3 (7)	0.1696	1.49 × 10^−2^	*METTL11B*	α N-terminal protein methyltransferase 1B	Proteins methylation
chr12:119772354-119772577	5 (5)	0.1682	1.49 × 10^−2^	*CCDC60*	Coiled-coil domain-containing protein 60	Muscular dystrophy type A6, Neuronitis
chr4:81117647-81119473	14 (20)	0.1638	2.86 × 10^−3^	*PRDM8*	PR domain zinc finger protein 8	Transcription regulation, Histone methyltransferase; Progressive myoclonic epilepsy-10
chr2:159651813-159651918	2 (4)	0.1618	3.09 × 10^−2^	*DAPL1*	Death-associated protein-like 1	Apoptosis, Early epithelial differentiation
chr11:128554939-128557589	13 (19)	0.1618	8.00 × 10^−3^	*FLI1*	Friend leukemia integration 1 transcription factor	Transcription factor; Hematopoiesis; Hemorrhagic diseases, Bleeding disorder platelet-21
chr6:33043868-33044510	5 (13)	0.1564	9.71 × 10^−3^	*HLA-DPB1*	HLA class II histocompatibility antigen	Peptide antigen binding; Berylliosis, Granulomatosis with polyangiitis, Juvenile idiopathic arthritis
chr17:56744332-56744490	3 (3)	0.1551	1.49 × 10^−2^	*TEX14*	Inactive serine/threonine-protein kinase TEX14	Mitosis; Spermatogenesis; Spermatogenic failure; Azoospermia; Infertility
chr5:178422071-178422415	6 (11)	0.1546	3.54 × 10^−2^	*GRM6*	Metabotropic glutamate receptor 6	Signal transduction; Retinal dystrophy, Night blindness
chr17:7832680-7833237	9 (11)	0.1546	9.14 × 10^−3^	*KCNAB3*	Voltage-gated potassium channel subunit β-3	Signal transmission, Potassium ion transport; Cone-rod dystrophy-6
chr22:51016501-51017166	13 (16)	0.1527	1.49 × 10^−2^	*CPT1B*	Carnitine O-palmitoyltransferase 1, muscle isoform	β-oxidation pathway in muscle mitochondria; CPT I deficiency, Visceral steatosis

**Table 4:** A summary of the results of the differential DNA methylation analyses in three age groups of individuals with DS; the statistics on differentially methylated positions (DMPs), or CpG-sites, and differentially methylated regions (DMRs) are shown.

		Newborns	Toddlers	Adults
		(Muskens et al. 2021 [8])	(Current Study)	(Bacalini et al. 2015 [6])
DMPs	Total, *n*	652	4806	18,573
	Hypermethylated, %	48.9	82.0	65.0
DMRs	Total, *n*	1052	115	66
	Hypermethylated, %	48.0	83.5	73.0

**Table 5:** The 19 Down syndrome-associated differentially methylated regions (DMRs) identified across all three age groups of individuals with DS: newborns [8], toddlers (current study), and adults [6]. Genes reported as having pan-tissue DS-associated methylation signals [3] are marked in bold.

DMR Position (GRCh37/hg19)	CGI Relation	Gene Name	Gene Region	DNAME Difference in DS (Mean Delta-Beta)		
				Newborns	Toddlers	Adults
chr1:36786285-36787932	CGI	*SH3D21; FAM176B*	Gene Body	0.0843	0.1851	0.2014
chr2:54086854-54087343	CGI	*ASB3; GPR75*	5′UTR, TSS200	−0.1151	−0.1377	−0.2035
chr4:81117647-81119473	CGI	*PRDM8*	5′UTR, TSS1500	0.1739	0.1638	0.1975
chr4:145566200-145566903	CGI	*HHIP*	TSS1500	0.079	0.1312	0.163
chr5:176827082-176827697	CGI	*PFN3*	1stExon, TSS200	0.0447	0.1747	0.3039
chr6:31939106-31939546	CGI Shore	***STK19***; *DOM3Z*	5′UTR, TSS1500	0.0154	0.1315	0.1939
chr6:33282628-33282997	CGI	***TAPBP***; ***ZBTB22***	TSS1500	0.0355	0.1080	0.1845
chr6:44243304-44243750	CGI	*TMEM151B*	Gene Body	0.117	0.1598	0.1782
chr7:27142618-27143788	CGI	*HOXA2*	TSS1500	−0.0408	−0.1415	−0.2116
chr7:27169957-27171051	CGI	***HOXA4***	1stExon, 5′UTR, TSS200	0.0811	0.1421	0.2069
chr9:34370835-34371380	CGI	*MYORG*	Gene Body	0.0869	0.2016	0.2095
chr10:70321668-70322874	CGI Shore	*TET1*	5′UTR	−0.0449	−0.1486	−0.1820
chr12:119772354-119772577	CGI	*CCDC60*	1stExon, 5′UTR, TSS200	0.0721	0.1682	0.1778
chr13:113689776-113689728	CGI Shore	*MCF2L*	Gene Body	−0.1022	−0.1722	−0.2070
chr16:979488-979898	CGI	*LMF1*	Gene Body	−0.1657	−0.1698	−0.3292
chr16:2029256-2030892	CGI	*NOXO1*	Gene Body	0.0492	0.1415	0.2216
chr18:77905408-77905751	CGI	***PARD6G-AS1***	TSS200	−0.0868	−0.1317	−0.1846
chr21:36258423-36259797	CGI	***RUNX1***	1stExon, 5′UTR	0.2733	0.2812	0.3557
chr22:51016501-51017166	CGI	***CPT1B***	1stExon, 5′UTR, TSS200	0.2096	0.1527	0.2586

## Data Availability

DNA methylation datasets were deposed in the NCBI Gene Expression Omnibus repository under accession number GSE174555.

## Supplementary Materials

The following are available online at https://www.mdpi.com/article/10.3390/genes12081115/s1: Table S1: Individual distributions of blood cell types predicted based on the whole-blood DNA methylation data in toddlers with Down syndrome (DS) and typically developing peers (TD). Table S2: Differentially methylated positions, DMPs: HME450 probes that show a significant difference (a fold change of 1.2 and over at a *p*_adj_ < 0.05) in the average methylation level (beta-value) in the blood cells of toddlers with Down syndrome (DS) compared to typically developing peers (TD). Table S3: The distribution of DS-associated differentially methylated positions (DMPs) across autosomes and genomic regions and considering their distinctive methylation status in toddlers DS marked as hypermethylated (HyperME) and hypomethylated (HypoME). The corresponding distributions for the HME450 array are provided. Table S4: List of genes containing DS-associated differentially methylated CpG sites within their promoter-related regions. Table S5: Gene Ontology (GO) terms and Human Phenotype Ontologies (HPO) overrepresented in the set of genes differentially methylated in DS. Functional categories are ranked by *p*-values of the enrichment tests. Table S6: Genomic regions defined as differentially methylated regions (DMRs) in the blood cells of toddlers with Down syndrome (DS) compared to typically developing peers (TD). Table S7: DS-associated differentially methylated positions (DMPs), or CpG sites, in blood cells of individuals with Down Syndrome overlapping across different age groups: newborns, toddlers, and adults. Table S8: DS-associated differentially methylated regions (DMRs) in the blood cells of individuals with Down syndrome overlapping across different age groups: newborns, toddlers, and adults.
